# CXCL12 modulation of CXCR4 and CXCR7 activity in human glioblastoma stem-like cells and regulation of the tumor microenvironment

**DOI:** 10.3389/fncel.2014.00144

**Published:** 2014-05-28

**Authors:** Roberto Würth, Adriana Bajetto, Jeffrey K. Harrison, Federica Barbieri, Tullio Florio

**Affiliations:** ^1^Sezione di Farmacologia, Dipartimento di Medicina Interna, University of GenovaGenova, Italy; ^2^Centro di Eccellenza per la Ricerca Biomedica, University of GenovaGenova, Italy; ^3^Department of Pharmacology and Therapeutics, College of Medicine, University of FloridaGainesville, FL, USA

**Keywords:** CXCL12, CXCR4, CXCR7, glioblastoma, cancer stem cells

## Abstract

Chemokines are crucial autocrine and paracrine players in tumor development. In particular, CXCL12, through its receptors CXCR4 and CXCR7, affects tumor progression by controlling cancer cell survival, proliferation and migration, and, indirectly, *via* angiogenesis or recruiting immune cells. Glioblastoma (GBM) is the most prevalent primary malignant brain tumor in adults and despite current multimodal therapies it remains almost incurable. The aggressive and recurrent phenotype of GBM is ascribed to high growth rate, invasiveness to normal brain, marked angiogenesis, ability to escape the immune system and resistance to standard of care therapies. Tumor molecular and cellular heterogeneity severely hinders GBM therapeutic improvement. In particular, a subpopulation of chemo- and radio-therapy resistant tumorigenic cancer stem–like cells (CSCs) is believed to be the main responsible for tumor cell dissemination to the brain. GBM cells display heterogeneous expression levels of CXCR4 and CXCR7 that are overexpressed in CSCs, representing a molecular correlate for the invasive potential of GBM. The microenvironment contribution in GBM development is increasingly emphasized. An interplay exists between CSCs, differentiated GBM cells, and the microenvironment, mainly through secreted chemokines (e.g., CXCL12) causing recruitment of fibroblasts, endothelial, mesenchymal and inflammatory cells to the tumor, *via* specific receptors such as CXCR4. This review covers recent developments on the role of CXCL12/CXCR4–CXCR7 networks in GBM progression and the potential translational impact of their targeting. The biological and molecular understanding of the heterogeneous GBM cell behavior, phenotype and signaling is still limited. Progress in the identification of chemokine-dependent mechanisms that affect GBM cell survival, trafficking and chemo-attractive functions, opens new perspectives for development of more specific therapeutic approaches that include chemokine-based drugs.

## BACKGROUND

Chemokines (CKs) and their cognate receptors are constitutively expressed in the central nervous system (CNS) where they control complex physiological functions. In particular, the pleiotropic chemokine CXCL12 (formerly known as stromal-cell derived factor-alpha, SDF1-α) and its receptors CXCR4 and CXCR7 are key regulators in CNS development, and are involved in neuromodulation, neuroprotection, and control the interactions between neurons, microglia and astrocytes in adult brain (Rostene et al., 2007). Altered expression of CXCR4 and CXCL12 has also been associated to CNS diseases, such as HIV encephalopathy, stroke and multiple sclerosis, among others (Bajetto et al., 2001a; Rostene et al., 2011). A role for CXCL12 modulation of CXCR4 and CXCR7 was identified for most of the brain tumors including gliomas, meningiomas and even pituitary adenomas that frequently overexpress these receptors (Bajetto et al., 2007; Barbieri et al., 2007; Duda et al., 2011). In this review we analyze the role of CKs in glioblastoma (GBM) development, diffusion and recurrence.

GBM is the most common and most malignant primary glial tumor in adults, characterized by an invariably poor outcome and limited therapeutic options (Dolecek et al., 2012). Standard GBM management involves maximal surgical resection, followed by radiotherapy with concomitant and adjuvant chemotherapy with temozolomide, but in most cases GBM rapidly relapses. Available treatments at relapse are largely ineffective and median overall survival of GBM patients is about 15 months (Stupp et al., 2005).

There is increasing evidence that tumor development, growth, recurrence and resistance to chemo- and radio-therapy is related to the presence of a cell subpopulation, named cancer stem cells (CSCs), nowadays identified in different human hemopoietic and solid cancers, including GBM. Efficient CSC eradication represents the ineludible goal to prevent tumor relapse and thus a target for all new anticancer approaches.

Beside its functional expression in embryonic pluripotent stem cells, in adults CXCL12/CXCR4/R7 axis controls tissue-specific stem cell proliferation (Singh et al., 2013). Similar functions have been hypothesized to occur also in CSCs. Thus the definition of mechanisms and downstream mediators of CXCR4/R7 activation by CXCL12, in normal and malignant differentiated cells, their progenitors, and in normal and CSCs, is highly relevant for both cancer biology and perspective therapeutic targeting.

## CXCL12/CXCR4–CXCR7 SIGNALING

For many years CXCR4 has been considered the unique receptor for CXCL12 and CXCL12 the sole ligand for CXCR4, a singular exception in the CK family that usually shows promiscuous binding within multiple CKs and receptors. Later, CXCR7 (originally named RDC1) was identified as second receptor for CXCL12, showing 10-fold higher affinity for CXCL12 than CXCR4 (Balabanian et al., 2005), CXCR7 is a member of the atypical CK receptor subgroup (ACKR), also including DARC, D6, and CCRL1, that do not activate G-proteins after correct binding with the respective cognate ligand (Bachelerie et al., 2014). ACKR3 has been proposed as the acronym for CXCR7 in this new nomenclature system. Interestingly, CXCL12 shares CXCR7 binding with another CK, CXCL11 (interferon-inducible T-cell α chemoattractant, ITAC) that is also a ligand for CXCR3 (Singh et al., 2013).

In the CXCR7 amino acid sequence, the highly conserved DRYLAIV domain, which controls G-protein binding and activation, is DRYLSIT (Thelen and Thelen, 2008). Typical CK responses, mediated by G protein activity, such as intracellular Ca^2^^+^ mobilization or modulation of adenylyl cyclase activity, are not generated after CXCR7 binding. Due to the absence of Gi-coupling, CXCR7 was initially proposed to be a decoy receptor, acting as a CXCL12 (and CXCL11) scavenger and able to promote ligand internalization and degradation, to reduce CXCR4 activity (Graham et al., 2012). However, the current vision is that this represents only one of the possible mechanisms by which CXCR7 modulates cellular functions (Sanchez-Martin et al., 2013). Emerging evidence suggests that CXCR7 can activate intracellular signaling pathways, and in particular it is able to elicit Akt, MAP kinase (MAPK), and Janus kinase-signal transducer and activator of transcription (JAK/STAT3) activation, either by direct modulation, through a β-arrestin-dependent pathway (Singh et al., 2013) or after heterodimerization with CXCR4 (Wang et al., 2011; Hattermann and Mentlein, 2013).

CXCL12 is a homeostatic CK, which controls hematopoietic cell trafficking and adhesion, in immune surveillance and development, being constitutively expressed in different organs (e.g., bone marrow, heart, liver, lung, lymph nodes, liver, brain, kidney, pituitary, among others). However, CXCL12 production has been also correlated with pathological processes, such as inflammation, heart failure, cell damage after organ irradiation or during chemotherapy. In particular, CXCL12 secretion is particularly relevant in hypoxic and pro-angiogenic environments within tumors or during autoimmune diseases (Li and Ransohoff, 2009). CXCR4 is also a rather ubiquitous receptor, with a relevant role at the level of endothelial mature and precursor cells and pericytes in healthy conditions and in hypoxic or damaged vascular tissues, including injured carotid arteries and atherosclerotic plaques (Petit et al., 2007).

**FIGURE 1:**
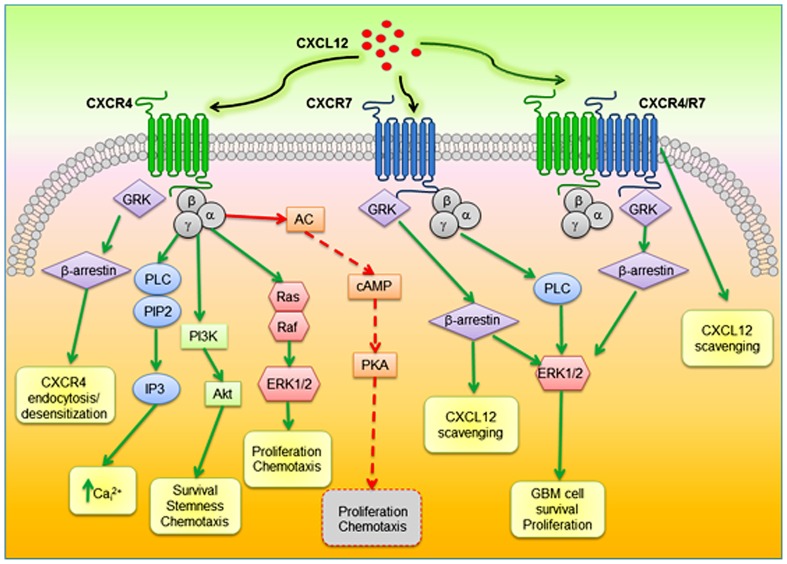
**Schematic diagram of proposed CXCR4–CXCR7 crosstalk affecting major signaling pathways related to cell survival, proliferation, and migration.** CXCL12 binds to CXCR4 and CXCR7, which can form homodimers or heterodimers. CXCR4–CXCR7 heterodimerization induces a conformational change of CXCR4/G-proteins and blocks signaling. CXCL12–CXCR4 interaction activated by CXCL12 triggers GPCR signaling through PI3K/Akt, PLC/IP3, and ERK1/2 pathways, and mobilization of Ca^2^^+^ from endoplasmic reticulum *via* inhibition of adenyl cyclase mediated cAMP production, thus regulating cell survival, proliferation, and chemotaxis. Beta-arrestin pathway can be activated through GRK to internalize CXCR4. When CXCR7 binds CXCL12, activation of the β-arrestin may lead to scavenging of CXCL12. In glioblastoma CXCL12/CXCR7 also controls cell survival through ERK1/2. AC, adenylyl cyclase; PLC, phospholipase C; PIP2; phosphatidylinositol 4,5-bisphosphate; IP3, inositol 1,4,5 trisphosphate; PI3K, phosphoinositide-3 kinase; ERK1/2, extracellular regulated kinase 1/2; GRK, GPCR kinase

CXCL12 binding to CXCR4 triggers receptor homo- and heterodimerization, often, but not always, with CXCR7, depending on the co-expression level of receptors (Levoye et al., 2009). Ligand binding changes the CXCR4 three-dimensional conformation favoring heterotrimeric G-proteins GDP/GTP exchange and dissociation into α- and βγ-subunits, that, in turn, activate multiple transductional pathways (Bajetto et al., 2001a): α_i_ subunits inhibit cAMP formation *via* modulation of adenylyl cyclase activity; the α_q_-subunit activates the phospholipase C (PLC)-β, which hydrolyzes PIP2 (phosphatidylinositol 4,5-bisphosphate) inducing the generation of diacylglycerol (DAG) and inositol 1,4,5 trisphosphate (IP3) that controls the release of intracellular Ca^2^^+^ from ER and the activation of protein kinase C; Gαi subunits also induce the activation of the transcription factor nuclear factor-κB (NF-κB), the Ca^2^^+^-dependent tyrosine kinase PYK2, JAK/STAT, and the activation of the phosphoinositide-3 kinase (PI3K)-Akt pathway, leading to cell survival and proliferation. The βγ dimer, acting as a functional subunit, is involved in Ras activation of ERK1/2 MAPK cascade, leading to changes in gene expression and cell cycle progression. CXCR4 also regulates cell survival by the G protein-dependent activation of JNK and p38 MAPKs. Further, βγ dimers interact with ion channels and activate PI3K, modulating CXCL12-dependent chemotaxis. CXCL12 also causes CXCR4 desensitization and uncoupling from G-proteins by GPCR kinase (GRK)-dependent phosphorylation and subsequent interaction of CXCR4 with β-arrestin that mediates internalization of the receptor (Cheng et al., 2000) and targets desensitized CXCR4 to clathrin-coated pits for endocytosis. Moreover, interactions between CXCR4 and β-arrestin also promote the activation of downstream intracellular mediators including MAPKs (p38, ERK1/2) and CXCL12-dependent chemotaxis (Sun et al., 2002). Cell migration is directed by CXCR4 by the formation of a CK gradient controlled by internalization of CXCL11 or CXCL12 bound to CXCR7, without the generation of intracellular signaling (Luker et al., 2009). The formation of CXCR4–CXCR7 heterodimers, modulates CXCR4 signaling (Levoye et al., 2009) and enhances CXCL12-dependent intracellular Ca^2^^+^ mobilization and ERK1/2 phosphorylation (Sierro et al., 2007), while chemotaxis induced by CXCL12 binding to CXCR4 is blocked by CXCR7 when expressed in the same cells (Decaillot et al., 2011). The enhanced activity of CXCR4–CXCR7 heterodimers in recruiting a β-arrestin complex, provides mechanistic insight into the growth, survival, and migratory advantage provided by CXCR4 and CXCR7 co-expression in cancer cells. β-arrestin recruitment to the CXCR4/CXCR7 complex enhances downstream, β-arrestin-dependent cell signaling (ERK1/2, p38, SAPK/JNK), which induces cell migration in response to CXCL12 (Cheng et al., 2000; Sun et al., 2002; Singh et al., 2013). CXCR7 monomers also promote ERK1/2 phosphorylation and nuclear translocation via G-protein-independent, β-arrestin-mediated signaling (Rajagopal et al., 2010; Decaillot et al., 2011). CXCR7 mediates CXCL12 signaling in cultured cortical astrocytes and Schwann cells that co-express CXCR4. Stimulation of astrocytes with CXCL12 activates ERK1/2, Akt but not p38 which was still evident after gene silencing of CXCR4 but fully abrogated by depletion of CXCR7. Conversely, in Schwann cells CXCL12 triggers also p38 phosphorylation altogether with ERK1/2 and Akt, but these effects require the activation of both receptors (Odemis et al., 2010). A diagram of intracellular transduction pathways related to CXCR4 and CXCR7 activation is depicted in **Figure 1**.

The interaction of CXCR7 with CXCL11 further complicates this chemokinergic system since CXCL11 also binds CXCR3, to induce either proliferative or growth inhibitory signals, depending on the CXCR3 variant (A or B; Singh et al., 2013). Moreover, besides CXCL11, CXCR3 is also bound by CXCL9 and CXCL10 to promote tumor growth, metastasis, angiogenesis and immune cell infiltration into tumors. GBM expression of CXCR3 was confirmed in human and murine GBM cell lines and its activation promotes proliferation *in vitro* and experimental tumor progression *in vivo* (Liu et al., 2011). The biological effects of the above described CK-receptor interactions is strictly related to receptor affinity, crosstalk of shared ligands, and associated intracellular signaling in both normal and tumor cells.

## MULTIPLE ROLES OF CXCL12/CXCR4-R7 NETWORK: REGULATION OF EARLY DEVELOPMENT OF THE CNS

CKs are pivotal regulators of cell migration, adhesion, and proliferation not only during inflammation and immune surveillance but also during CNS development. In particular, beside inflammatory or homeostatic leukocyte migration, CXCL12 retains a primordial role, highly conserved through the evolution, in the regulation of embryonic and adult stem cell directional migration. The first evidence of the function of CXCL12 in neural development was suggested by the lethal phenotype of CXCR4- and CXCL12-knockout mice (Ma et al., 1998; Zou et al., 1998), both exhibiting abnormal neuronal migration in the cerebellum, dentate gyrus and dorsal root ganglia, in addition to defective lympho-myelopoiesis, and imperfect vasculature and heart development. During cerebellar development CXCR4-positive granule cell precursors are retained in the external granule layer through their interaction with CXCL12 expressed in the overlying pial meninges, ensuring sufficient cell proliferation and allowing the migration to the internal granule layer only when the cerebellar cortex is ready to receive them. Altered CXCL12/CXCR4 interaction causes a premature migration of granule precursors and disorganized layer formation (Stumm et al., 2003; Huang et al., 2014). CXCL12 anchors cerebellar granule precursors and favors their proliferation synergistically with Sonic hedgehog (Shh). The CXCL12/CXCR4 axis also controls the tangential migration of post-mitotic neurons (Tiveron et al., 2006).

Thus, CXCL12/CXCR4 signaling regulates the development of many structures in the central and peripheral nervous system, including cerebellum, cortex, hippocampus and dorsal root and sympathetic ganglia, controlling migration and positioning of cerebellar granule cells, Cajal–Retzius cells, cortical interneurons, and hindbrain pontine neurons (Li and Ransohoff, 2009; Zhu et al., 2009; Zhu and Murakami, 2012). CXCL12/CXCR4 regulation of stem cell functioning continues also in adults, in neurogenic niches of brain and bone marrow. Hematopoietic progenitors cells (HPCs) are retained in bone marrow through a CXCR4/CXCL12 interaction that regulates also HPCs homing to this niche after transplantation (Kaplan et al., 2007) survival and proliferation. Interestingly, a similar mechanism has been demonstrated in adult neural progenitor cells (NPCs) or neural stem cells (NSCs; Li and Ransohoff, 2009).

First detected in the 1960s (Altman, 1962), two main areas have been identified in adult mammalian brain where NSCs are localized: the subventricular zone (SVZ) along the lateral walls of the lateral ventricles, and the subgranular zone (SGZ) in the dentate gyrus of hippocampus. NSCs have the potential to self-renew, proliferate, and differentiate into neurons, astrocytes, and oligodendrocytes (Laywell et al., 2007), also in response to ischemic or hypoxic insults (Yang and Levison, 2006; Laywell et al., 2007; Miles and Kernie, 2008; Jin-qiao et al., 2009).

In postnatal brain, CXCR4 expression continues in NPCs in SVZ of lateral ventricle and SGZ of dentate gyrus, the adult brain neurogenic areas, while CXCL12 is expressed in ependymal and endothelial cells adjacent to the proliferating areas. CXCL12 triggers the homing of SVZ NSCs from the ependymal niche to the vascular niche that is abrogated by CXCR4 knockdown. Notably, it has been suggested that the migration of responsive cells to the vascular niche, or non-migration of resting cells in the ependymal niche, is regulated by the levels of CXCL12 in the microenvironment (Miller and Gauthier-Fisher, 2009).

Thus, both in adult and embryonic brain, one of the main physiological roles of the CXCL12/CXCR4 axis is the positioning of NPCs near blood vessels or meninges that provide important source of factors for proliferation and differentiation. Generally, CXCR7 has a more limited and less characterized pattern of expression than CXCR4, and it is primarily involved in vascular and cardiac development, rather than hematopoiesis, as observed *in vivo* in CXCR7 knockout mice that die at birth from severe heart and vascular defects (Sierro et al., 2007). However, CXCR7 is also expressed during mouse embryogenesis in the neural tube and brain concomitantly with neural crest development and vascularization. (Schonemeier et al., 2008b). During rat brain development, CXCR7 expression starts at E11.5, increases between E15 and E18 in the marginal zone/layer I, and decreases postnatally. In the cerebral cortex, CXCR7 is expressed in GABAergic neuron progenitors, Cajal–Retzus cells, and neural precursors (Schonemeier et al., 2008a).

The molecular function of CXCR7 within the brain has been investigated by studies in zebrafish that provided the first and strong evidence that CXCR7 acts as scavenger receptor, mediating CXCL12 internalization and providing directional cell migration of primordial germ cells to the gonads and the formation of the posterior lateral line. CXCR4 is expressed by migrating cells and CXCR7 acts by sequestrating CXCL12 from non-target areas, allowing the correct cell migration (Dambly-Chaudiere et al., 2007). In the absence of CXCR7, migrating cells still respond to CXCL12, but their movement ends in undesirable sites because of a specific accumulation that prevents the formation of a CXCL12 gradient required for a directional migration. (Boldajipour et al., 2008; Cubedo et al., 2009). In mammals, the scavenger function of CXCR7 has been established in mouse heart valve, as well as umbilical vein endothelial cells (Naumann et al., 2010) and it was shown to be responsible of interneuron migration. Accordingly, CXCR4 and CXCR7 are co-expressed in migrating interneurons but they have a different subcellular localization: CXCR4 in the plasma membrane and CXCR7 in intracellular recycling endosomes (Wang et al., 2011; Sanchez-Alcaniz et al., 2011). CXCR7 is frequently expressed, in the absence of CXCR4, in forebrain in postnatally generated olfactory interneuron precursors, further demonstrating that CXCR7 is an independent, direct mediator of CXCL12 signaling (Tiveron et al., 2010).

The relevance of CXCL12 and CXCR4/R7, in CNS functions and brain development is even more important considering the consequences of the cancer stem cell hypothesis (Singh et al., 2003, 2004a), based on the concept that tumors derive from cells endowed with stem or stem-like features in which alterations of the self-renewal mechanisms are induced. Therefore, understanding the CK-dependent mechanisms associated with the stemness in normal neural progenitors might help to clarify their activity in cancer development.

## ROLE OF CXCL12–CXCR4/R7 IN GLIOBLASTOMA

CXCL12–CXCR4/R7 system plays a central role in tumor development and tumor cell proliferation, mainly acting *via* an autocrine/paracrine mechanism, and contributes to the dissemination and invasiveness of several human cancers, including pancreatic, colon, ovarian, prostate, breast, and renal carcinomas, lymphoma, melanoma, neuroblastoma and GBM (Zlotnik, 2006; Barbieri et al., 2010; Lippitz, 2013; Singh et al., 2013). Moreover, less malignant or benign tumors (i.e., pituitary adenomas, meningiomas, etc.) seem to be regulated by the activity of this chemokinergic axis (Bajetto et al., 2007; Barbieri et al., 2008; Wurth et al., 2011).

Additionally, CXCR4 expression in tumor cells was associated with metastasis of many human malignancies (Muller et al., 2001; Ben-Baruch, 2008; Zlotnik, 2008; Ferrari et al., 2012) favoring their migration and homing toward CXCL12 expressing organs (lung, liver, brain, lymph nodes, bone marrow; Teicher and Fricker, 2010).

The significance of the expression and function of the CXCL12/CXCR4 axis in brain tumors has been intensely investigated in adult and children GBM, astrocytoma, medulloblastoma, oligodendroglioma, and oligodendroastrocytoma (Domanska et al., 2011). In GBM, CXCR4 and CXCL12 are overexpressed in tumor tissue when compared with normal adjacent parenchyma, and their expression level is correlated with tumor grade and poor prognosis (Salmaggi et al., 2005a; Bian et al., 2007). Immunohistochemical studies showed that in GBM CXCR4 and CXCL12 expression does not co-localize with tumor proliferating cells (identified by MIB-1 expressing cells) but they are both mainly localized in hypoxic regions, characterized by necrosis (Rempel et al., 2000; Salmaggi et al., 2005b; Zagzag et al., 2008). In these perinecrotic areas (“pseudopalisading” necrosis), characterized by high cellularity (Rempel et al., 2000; Bajetto et al., 2006; Rong et al., 2006) due to the powerful invasion of glioma cells (Sciume et al., 2010), CXCR4 and CXCL12 co-localize in the same tumor cells (Bajetto et al., 2006). Pseudopalisade areas are peculiar pathologic structures of GBM resulting from a sequence of vascular occlusion and hypoxia (Brat and Van Meir, 2004) leading to migration and accumulation of GBM cells around central necrotic areas and microvascular hyperplasia induced by hypoxic pseudopalisading cells (Jin et al., 2006). Hypoxia promotes GBM angiogenesis, not only *via* hypoxia-inducible factor-1α (HIF-1α) that directly induces the transcription of VEGF and cytokines (i.e., TNF-α), and stimulates CXCL12 expression, but also up-regulates CXCR4 expression in pseudopalisades. Thus, CXCL12 drives angiogenesis either directly or in a paracrine manner, supporting tumor growth and GBM cell migration far from hypoxic pseudopalisades, allowing for both necrotic area formation and peripheral invasiveness of GBM.

On the other hand, CXCR4 and CXCL12 expression frequently occurs in GBM proliferating vascular endothelium, but not in endothelial cells from astrocytomas in which proliferation of microvessels is less abundant (Bajetto et al., 2006).

Interestingly, the mechanism of dissemination of glioma cells within the brain, differently from other cancers, does not occur through lymphatic and hematogeneous spread. GBM cells invade the adjacent brain parenchyma with a morphological pattern known as “Scherer’s structures” that include normal brain structures (white matter, blood vessels, and parenchyma) where CXCL12 is highly expressed. GBM cells, organized around neuron and blood vessels in subpial regions and in white matter express high levels of CXCR4: VEGF-dependent CXCL12 up-regulation in neuronal and endothelial cells induces the migration of CXCR4-positive GBM cells, representing the molecular mechanisms of Scherer’s structure formation (Ehtesham et al., 2006; Zagzag et al., 2008; Munson et al., 2013). CXCL12 exerts also pro-angiogenic activity, recruiting CXCR4-positive, circulating bone marrow-derived cells (Petit et al., 2007) and promoting tumor vasculature recovery after irradiation, as a consequence of treatment-induced hypoxia and HIF-1α activation, which results in increased CXCL12 expression (Jin et al., 2006; Kioi et al., 2010).

The ability of CXCL12 to guide GBM cell migration has been widely supported by *in vitro* experiments (Rubin et al., 2003; Bajetto et al., 2006) and, although the molecular mechanisms involved are not definitively identified, the effect of CXCL12 results from a functional cooperation with EGFR and PDGFR, overexpressed in GBM cells (Woerner et al., 2005; Sciaccaluga et al., 2013).

The tumor microenvironment, consisting of constitutive non-cancerous cells (fibroblasts, endothelial cells, and immune cells), as well as connective tissue and extracellular matrix, contributes to GBM (and other solid tumors) development, proliferation, invasiveness and angiogenesis (Domanska et al., 2013). CXCL12/CXCR4 axis acts on the tumor microenvironment through the modulation of the expression and secretion of other CKs (i.e., CCL2, CXCL8) and, concomitantly, CXCR4 expression could be influenced by cytokines (TNFα, IFNγ, IL4-6-10) produced by cells in the microenvironment. These interactions represent an indirect mechanism mediating CXCL12/CXCR4-dependent promotion of survival, proliferation and migration of tumor cells (Zhou et al., 2002; Burger and Kipps, 2006).

Hypoxia enhances CXCL12 secretion in cancer-associated fibroblasts which in turn feeds tumor development either by direct stimulation of tumor cells expressing CXCR4 (paracrine effect) or recruiting endothelial cells for angiogenesis (endocrine effect; Burger and Kipps, 2006). Stromal fibroblasts support the growth of neoplastic cells through elevated secretion of CXCL12 (Orimo et al., 2005), and integrins induce expression of CXCR4 and growth-factor receptors sustaining a pro-survival loop for tumor cells.

The interaction of GBM cells with the microenvironment that protects cancer cells from the chemo- and radio-therapy stress, becomes even more relevant in the context of CXCL12/CXCR4 up-regulation observed after treatment with anticancer drugs, and particularly after anti-VEGF antibodies (Shaked et al., 2008; Kioi et al., 2010; Keunen et al., 2011).

Several studies investigated the signal transduction of the CXCL12/CXCR4 axis in normal glial cells or in cell lines derived from human GBMs, being the expression of ligand and receptor almost always reported. CXCR4 and CXCL12 expression was described in primary cultures of rat type I astrocytes, cortical neurons and cerebellar granule cells and treatment with CXCL12 induced proliferation of normal astrocytes through the activation of ERK1/2 and PI-3K pathways (Bajetto et al., 1999a, b, 2001b). In human GBM cell lines (U87-MG, DBTRG-05 and A172), CXCL12 that is released in the extracellular medium, supports cell growth, likely through an autocrine/paracrine mechanism by the activation of intracellular ERK1/2 and Akt pathways (Barbero et al., 2002, 2003). However, differently from normal astrocytes, GBM cell lines show constitutive Akt activation, further increased by CXCL12, and ERK1/2 and Akt are independently involved in cell proliferation. Conversely, the glioma onco-suppressive gene LRRC4 inhibits CXCL12/CXCR4-induced cell proliferation, chemotaxis and invasiveness reducing ERK1/2 and Akt signaling (Wu et al., 2008).

*In vivo* studies, in which GBM cells are intracerebrally implanted, showed that CXCL12/CXCR4 binding activates matrix metalloproteinases (MMPs) that contribute to the infiltrative behavior of GBM cells within the brain parenchyma (Zhang et al., 2005).

While CXCL12/CXCR4 activation within both cancer cells and local stroma clearly contributes to GBM cell proliferation, spreading, and survival to therapy, more recent studies demonstrated that CXCR7 is an alternative, or additional, regulator of GBM growth. CXCR7 is up-regulated in all pathological conditions in which CXCL12 activity is enhanced, including neoplastic diseases, and contributes to tumor growth, adhesion, survival, angiogenesis, and invasion of breast, lung and prostate carcinomas (Miao et al., 2007; Wang et al., 2008) and promotes tumor development in mice (Burns et al., 2006). CXCR7 is highly expressed in tumor endothelial, microglial, and GBM cells (Hattermann et al., 2010). CXCR7 controls tumor diffusion through CXCL12 gradients and it is frequently detected in GBM-associated vasculature (Liu et al., 2010). The increase of CXCR7 expression in microvascular endothelial cells during hypoxia (Schutyser et al., 2007; Monnier et al., 2012) favors CXCL12-induced glioma cell migration (Esencay et al., 2013) facilitating the binding of CXCL12 to endothelial cells (Burns et al., 2006; Liu et al., 2010; Dai et al., 2011) and the activation of CXCL12-mediated cell crossing through endothelium (Mazzinghi et al., 2008; Zabel et al., 2009; Dai et al., 2011).

## CXCL12, CXCR4–CXCR7 ACTIVITY IN HUMAN GLIOBLASTOMA STEM-LIKE CELLS

**FIGURE 2:**
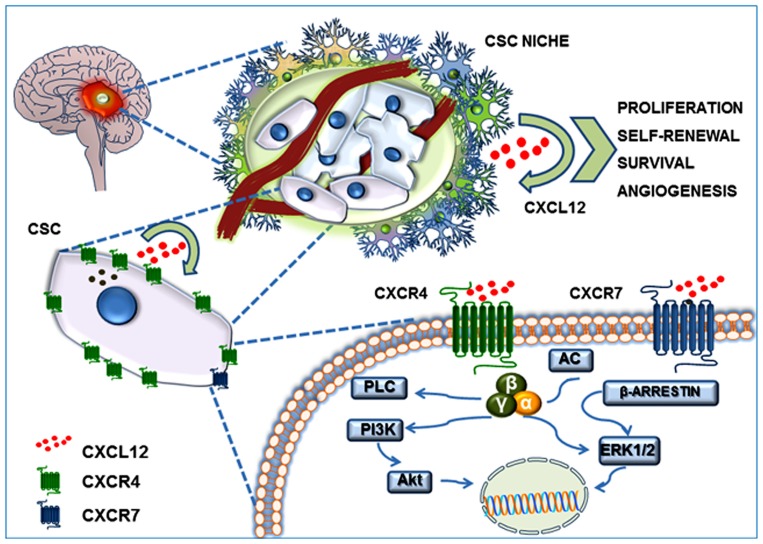
**CXCL12/CXCR4–CXCR7 system in the GBM CSC niche.** GBM CSC niche is a discrete microenvironment within the tumor mass. It is composed of a heterogeneous cell population that generally includes blood vessels, tumor cells, CSCs, extracellular matrix components, and a gradient of soluble factors. CXCL12 secretion by endothelial cells, tumor cells, and CSCs generates an autocrine and paracrine action which contributes to self-renewal, survival and migration of CSCs themselves, triggering Akt and MAP-kinase(s) intracellular signaling.

In recent years, the CSC theory has gained more experimental validation in addition to the refined theoretical definition. In particular, GBM is the tumor histotype that more precisely matches CSC criteria, in terms of heterogeneity and hierarchical organization of cells, identification of stem cell features in tumor cell subpopulations and, importantly, as far as pharmacological responses. Thus, considering the significant role of CXCL12–CXCR4/R7 axis in normal stem cell biology, it is evident that this chemokinergic system could play a relevant role in GBM CSCs. Moreover, according to genotypic and phenotypic evidence of a more close reproduction *in vitro* of the *in vivo* tumor characteristics of cultures enriched in CSCs, as compared with established cell lines (Lee et al., 2006), recent studies addressed the role of CXCL12 and its receptors in this GBM cell subpopulation (**Figure 2**).

### GLIOBLASTOMA CANCER STEM CELLS

Glioblastoma is a complex, heterogeneous tissue characterized by the coexistence of several different cell populations with a hierarchical organization. Among them, a relatively small population exhibits stem-like features, including the capacity to persist in a constant number through asymmetric mitotic cell division (self-renewal capacity), thus representing a drug-resistant cell reservoir to generate differentiated cells (multilineage differentiation potential), that in GBM are represented by differentiated progeny expressing either neuronal or glial markers. After the identification of distinctive stem cell markers, these cells were named cancer stem cells (CSCs). Importantly, besides showing NSC properties, CSCs are tumorigenic, phenocopying the original tumor when xenotransplanted in animal models. For this reason, they are also called tumor-initiating cells (TICs), in order to highlight their tumorigenic potential (Florio and Barbieri, 2012). CSCs have been detected in different hematological and solid tumors (Bonnet and Dick, 1997) and the first evidence supporting the presence of CSCs in GBM was reported in 2003 (Singh et al., 2003; Singh et al., 2004b). Several groups have subsequently described the isolation and characterization of GBM CSCs (Hemmati et al., 2003; Bao et al., 2006a; Liu et al., 2006; Bajetto et al., 2013; Griffero et al., 2009). Initially, the phenotypic characterization of GBM CSCs was based on the recognition of distinctive NSC markers, such as nestin, Sox2, Nanog, Oct4, BMI1, musashi-1, but later on, components of pathways active in brain development were found to be expressed in GBM CSCs, including Notch (Wang et al., 2010a), Wnt (Jin et al., 2011), bone morphogenetic protein (BMP; Piccirillo et al., 2006) and TGF-β (Ikushima et al., 2009). The five-transmembrane domain glycoprotein CD133 (prominin-1), initially reported as one of the most reliable markers to identify GBM CSCs (Beier et al., 2007), although conflicting reports were subsequently published and not labeling all CSC subpopulations, is still considered a key component of CSCs (Grosse-Gehling et al., 2013) acting as a regulator of cell survival inducing PI3K-Akt activation, *via* a direct interaction with p85 (Wei et al., 2013).

Conceivably, the plastic phenotype recently proposed for CSCs, rather than unique cellular marker(s), is the most valid hypothesis, also taking into account GBM cell heterogeneity and the high degree of plasticity that favors its aggressive behavior (Florio and Barbieri, 2012; Tang, 2012; Ruiz-Ontanon et al., 2013; Wurth et al., 2014).

The origin of GBM CSCs is still unclear and controversial. CSCs were proposed to derive from normal NSCs after the accumulation of oncogenic mutations and/or following events mediated by the microenvironment (Calabrese et al., 2007; Hjelmeland et al., 2011). More recently, it was suggested that differentiated neurons or astrocytes can be dedifferentiated and transformed, acquiring CSC-like features to originate histologically different GBMs (Friedmann-Morvinski et al., 2012).

Niches are the specific sites where normal stem cells reside and in adult tissues constitute a spatially distinct microenvironment, which contains stromal cells, blood vessels and high concentrations of extracellular matrix proteins and growth factors. The interaction between stem cells and specific niches is critical for the maintenance of their functional properties, and, in particular, for the balance between self-renewal and differentiation that regulates cell number and tissue homeostasis (Ramasamy et al., 2013).

Like NSCs, GBM CSCs require specific niches in which a permissive environment, ensuring the correct combination of supporting cells (endothelial cells, reactive astrocytes, pericytes, tumor-associated macrophages) and extrinsic factors, is essential for their maintenance and regulation.

There are relevant similarities between NSC and CSC niche:

#### Vasculature

Blood vessels are an integral component of both neural and cancer stem cell niches. Endothelial cells (EC) modulate NSCs not only by providing oxygen and nutrients, but also through regulation of their capacity to self-renew, proliferate and differentiate (Shen et al., 2004; Charles and Holland, 2010). A comparable and intricate relationship occurs in the niche where CSCs are closely connected to ECs establishing a paracrine modulation between these two cell types, largely mediated by the secretion of VEGF and CXCL12 (Calabrese et al., 2007; Yao et al., 2013). GBM CSCs not only release growth factors that induce proliferation of ECs (Bao et al., 2006b) but also may be themselves a direct source of angiogenesis by trans-differentiation into endothelial-like cells. On the other hand, ECs maintain CSCs in undifferentiated state, promote their tumorigenicity (Calabrese et al., 2007) and, *via* the interaction CXCL12–CXCR4, maintain GBM CSCs localized in the perivascular niche (Cheng et al., 2013).

#### Molecular signaling pathways

Developmental pathways (Notch, Wnt, Shh), and receptors activated by fibroblast growth factor (FGF), epidermal growth factor (EGF), transforming growth factor (TGF)- β, and CKs, primarily CXCL12, identified within the niche to maintain NSCs during development and in adult brain, are also involved in CSC survival (Gatti et al., 2013; Penuelas et al., 2009). The interaction between these signal transduction pathways results in self-renewal, sustained proliferation, and increased survival, invasiveness and drug resistance of CSCs (Mimeault et al., 2007).

### CXCL12/CXCR4-R7 AXIS IN GBM CSC REGULATION

As previously detailed, CXCL12 modulates tumor cell proliferation, angiogenesis and metastasis, acting as an autocrine/paracrine growth factor (Barbero et al., 2003; Barbieri et al., 2008; Pattarozzi et al., 2008), representing a promising target for the treatment of neoplasia. The concept of CSCs and their identification in several tumors highlights possible new roles for the CXCL12–CXCR4/R7 axis in tumor biology. CXCR4 (over)expression has been detected in CSCs derived from various of cancer histotypes, including pancreatic (Hermann et al., 2007), colon (Zhang et al., 2012), lung (Jung et al., 2013), breast (Dubrovska et al., 2012b) prostate (Dubrovska et al., 2012a), renal (Gassenmaier et al., 2013), and GBM (Singh et al., 2004b). Moreover, the recent demonstration that CXCR7 can also serve as an active receptor for CXCL12 (Odemis et al., 2012) has increased the interest for this chemokinergic system in CSC-related research.

Preclinical studies addressing the role of CXCL12 in GBM CSCs are listed in **Table 1** (generated from PubMed, using “CXCL12–CXCR4–CXCR7-GBM-CSCs” as keywords).

**Table 1:** CXCL12/CXCR4–CXCR7 axis in GBM CSC, a review of the literature.

CSC isolation	CSC characterization	CSC culture conditions	CXCL12 role	CXCR4–CXCR7 expression	Intracellular pathways	Reference
Primary patient-derived GBM cells	*In vitro* marker expression (nestin), *in vivo* tumorigenicity	Stem cell-permissive medium	Proliferation	High expression of CXCR4 in CSCs		Salmaggi et al. (2006)
Rat glioma cell line (C6)	*In vitro* marker expression (nestin), *in vivo* tumorigenicity	Stem cell-permissive medium (PDGF in place of EGF)	Angiogenesis	CXCR4^+^ CSCs		Folkins et al. (2009)
Primary patient-derived GBM cells	*In vitro* expression of nestin, CD133, Bmi-1, Sox2, Musashi-1, differentiation potential, and *in vivo* tumorigenicity	Stem cell-permissive medium	Proliferation	Overexpression of CXCR4 in CSCs		Ehtesham et al. (2009)
Primary patient-derived GBM cells	*In vitro* marker exression (CD133), *in vivo* tumorigenicity	Stem cell-permissive medium	*In vivo* proliferation and *in vitro* migration	Overexpression of CXCR4 in CSCs	AMD3100-sensitive CXCL12 activation of Akt and ERK1/2	Schulte et al. (2011)
CD133-cell sorting of primary patient-derived GBM cells and of GBM cell lines	*In vitro* marker expression (nestin, CD133), multilineage differentiation, *in vivo* tumorigenicity	Stem cell-permissive medium	*In vitro* migration; *in vivo* tumor growth and angiogenesis	CXCR4 overexpression in CSCs, whose abolishment diminished *in vivo* tumorigenesis	CXCL12 activated Akt and ERK1/2	Ping et al. (2011)
CD15/CD133 or CD15/L1CAM cell sorting of primary patient-derived GBM cells	*In vitro* marker expression (Sox2 and Olig2), self-renewal, multilineage differentiation, *in vivo* tumorigenicity	Stem cell-permissive medium	*In vitro* and *in vivo* migration and angiogenesis	CXCR4^+^ CSCs		Cheng et al. (2013)
Primary patient-derived GBM cells	Slow cycling subpopulation	Stem cell-permissive medium	*In vitro* proliferation, migration, self-renewal and angiogenesis	Enrichment of CXCR4 and CXCR7 in the slow-cycling subpopulation		Liu et al. (2013)
Rat GBM cell line (RG2)	*In vitro* marker expression (Nestin, ABCG2, musashi, Oct4, Nanog)	90% DMEM contained 10% fetal bovine serum	*In vitro* and *in vivo* proliferation, *in vitro* self-renewal	CXCR4^+^ CSCs	Disruption of CXCR4 impaired Akt and ERK1/2 activation	Lee et al. (2013)
Primary patient-derived GBM cells	*In vitro* marker expression (nestin, CD133), multilineage differentiation potential, *in vivo* tumorigenicity	Stem cell-permissive medium	*In vitro* self-renewal, survival, and clonogenicity	Variable expression of CXCR4, minimal expression of CXCR7	CXCL12 activated Akt and ERK1/2	Gatti et al. (2013)
Syngeneic murine CSC line, established from adult mice NSC	*In vivo* tumorigenicity	Stem cell-permissive medium	*In vitro* and *in vivo* proliferation	CXCR4^+^ CSCs		Uemae et al. (2014)
CD133-cell sorting of primary patient-derived GBM cells	*In vitro* marker expression (CD133), multilineage differentiation potential, *in vivo* tumorigenicity	Stem cell-permissive medium	Self-renewal through CXCR7	Details about receptor expression not provided		Walters et al. (2014)

*Stem cell-permissive medium: serum-free medium (DMEM or DMEM-F12 or Neurobasal) with EGF, bFGF and B27, cells growth in non-adherent condition (spheres). When not mentioned, the expression of CXCR7 has not been evaluated.*

Criteria used to identify and maintain CSC cultures differ among the various studies, mainly because of the absence of absolute and uniform biomarkers and, different methods of CSC isolation and in vitro culture enrichment have been reported, making study comparisons difficult. Although putative GBM stem-like cells have been isolated as a subpopulation within established cell lines, the isolation of tumor cell subpopulations from human post-surgical explants, grown as non-adherent neurospheres in serum-free medium enriched with growth factors (Folkins et al., 2009), is currently considered the most reliable source of GBM CSCs (**Table 1**). Long-term passaging of cells in media containing high percentages of serum irreversibly modifies both the phenotype and genotype of the cells as compared to those present in the original tumor, favoring the selection of mutated cells more fit to *in vitro* growth (Lee et al., 2006; Wakimoto et al., 2012). Moreover, early passage GBM CSCs grown under serum free conditions better recapitulate the *in vivo* invasive characteristics of the parental tumor when grown as intracranial xenografts, thus making them a more suitable model system.

The selection of tumor cell subpopulations on the basis of the expression of specific biomarkers common to both NSCs and GBM CSCs (in particular CD133 and nestin), could be performed as an additional step. Culture conditions defined for the propagation of NSCs (serum-free medium containing EGF and bFGF) are effective to sustain GBM CSC growth *in vitro,* allowing cells to grow as floating spheroids (neurospheres) that retain the stem cell phenotype. Noteworthy, neurosphere formation in limiting dilution experiments is one of the main tools used to assess *in vitro* CSC self-renewal (Carra et al., 2013). Finally, CSC isolation has to be further verified by *in vitro/in vivo* analysis of functional properties, such as multilineage differentiation and, most importantly, tumorigenicity in animal models.

The CXCL12–CXCR4 axis was reported as a main regulator of GBM CSC biological features: self-renewal, proliferation, migration, angiogenesis, and chemo-and radio-resistance. Overexpression of CXCR4 was observed in GBM CSCs, which increased proliferation in response to exogenous CXCL12 (Ehtesham et al., 2009). However, this CK is also released by CSCs, suggesting an autocrine/paracrine signaling mechanism (Salmaggi et al., 2006; Gatti et al., 2013). CXCR4 activation in GBM CSCs, in combination with VEGF and HGF signaling pathways under hypoxia, is a key factor in determining NSC tropism toward gliomas (Zhao et al., 2008). Further corroboration of these findings came from studies showing higher CXCR4 expression in GBM CSC cultures than in the differentiated tumor cells obtained from the same culture (Ehtesham et al., 2009). These reports paved the way for further studies that revealed high heterogeneity in CXCR4 expression amongst CSC cultures derived from individual human GBMs (Liu et al., 2013). This observation was supported and highlighted in a recent study showing, in five different CSC cultures, that the distinctive properties of original GBM are retained *in vitro*, including CXCR4 expression and CXCL12 secretion, but were highly variably among cultures, with a general inverse relationship between receptor expression and ligand secretion levels (Gatti et al., 2013).

*In vitro,* GBM CSC proliferation was induced either by treatment with exogenous CXCL12 (Liu et al., 2013) or by the receptor activation induced by CXCL12 secreted by CSCs in an autocrine fashion (Uemae et al., 2014). This effect was mainly mediated by CXCR4, since it was reversed in the presence of the CXCR4 antagonist AMD3100 (Schulte et al., 2011; Gatti et al., 2013). On the other hand, it was observed that the autocrine effects of CXCL12 promote GBM CSC survival to a higher extent than proliferation: CXCR4 blockade by AMD3100 reduces CSC survival proportionally to the amount of spontaneously released of CXCL12 (Gatti et al., 2013). The ability of AMD3100 to impair colony formation induced by both exogenous and secreted CXCL12 in GBM CD133-positive cells further confirms the autocrine growth-stimulation effect of this CK in this subset of GBM cells (Ping et al., 2011).

The role of CXCL12/CXCR7 axis in GBM CSC biology was only recently investigated and, even if a definitive establishment of its role was not provided, strong evidence supports its involvement in GBM CSC maintenance and tumorigenicity. Pharmacological inhibition of CXCR7 post irradiation caused tumor regression, reduced tumor recurrence, and substantially prolonged survival in a rodent model of GBM, likely interfering with CSCs (Walters et al., 2014). Heterogeneous cell surface expression of CXCR4 and CXCR7, despite similar levels of corresponding mRNAs, was also observed in primary GBM cell cultures. Analysis of cultures enriched in CSCs determined increased percentage of CXCR4- and CXCR7-expressing cells suggesting that both receptors might regulate stem phenotype. Heterogeneous functional responses to CXCL12 are evident, with different roles in promoting *in vitro* cell growth, migration, spherogenesis, and tube formation in individual cultures (Liu et al., 2013). However, CXCR4^+^, CXCR7^+^, and CXCR4^+^/CXCR7^+^ cell subpopulations present in cell cultures are all tumorigenic (Lee et al., 2013; Liu et al., 2013).

Conversely, other studies reported that GBM CSCs do not express, or express at a low level, CXCR7 (Hattermann et al., 2010; Gatti et al., 2013). Moreover, upon GBM CSC differentiation, CXCR4 levels diminish while CXCR7 increases, suggesting a prevalent role for CXCR7 in differentiated GBM cells (Hattermann et al., 2010).

The role of CXCL12–CXCR4 axis in GBM CSCs was corroborated by *in vivo* studies. In particular, knocking down CXCR4 using RNAi or inhibiting CXCR4 function by AMD3100 in CSCs, impairs proliferation *in vivo*, effectively reducing tumor growth in two different xenograft models (Ping et al., 2011; Lee et al., 2013); similarly shRNA CXCL12 knock-down in CSCs inhibited tumor growth *in vivo* (Uemae et al., 2014).

### SELF-RENEWAL

Besides proliferation and survival, CXCL12/CXCR4 axis plays a significant role in maintaining CSC self-renewal. Self-renewal *in vitro* can be evaluated through sphere formation and clonogenicity assays. These tests were performed in two different models of GBM CSCs (Gatti et al., 2013; Lee et al., 2013) showing that exogenous CXCL12 promoted sphere formation, and that either the pharmacological blockade of CXCR4 by AMD3100 (Gatti et al., 2013) or silencing the receptor (Lee et al., 2013) suppressed CSC sphere-forming ability after serial *in vitro* passages. The role of CXCL12 in CSC sustained self-renewal was also supported by the observation that disrupting CXCR4 signaling reduces the expression of genes associated with self-renewal activity (i.e., Oct4 and Nanog; Lee et al., 2013). Similarly, CXCR7 inhibition by CCX771 powerfully affects CSC self-renewal (Walters et al., 2014). Taken together, these data suggest that maintenance of stemness of the CSC subpopulation represents a relevant function related to CXCL12 activity.

### MIGRATION

CXCL12 stimulated *in vitro* migration of CSCs in a dose-dependent manner and co-administration of AMD3100 inhibited peak chemotactic responses. Conversely, the same treatment resulted in minor effects in continuous glioma cell lines, and only in the presence of extremely high concentrations of AMD3100 caused a statistically significant inhibition of migration (Schulte et al., 2011). However, individual CSC cultures displayed heterogeneous responses to CXCL12 in cell migration experiments *in vitro*. In particular, CXCL12 induced AMD3100-sensitive cell migration only in a subset of CSC cultures tested (Liu et al., 2013).

### ANGIOGENESIS

CSCs are also responsible for the development of GBM microvasculature. Tumor microvessels were demonstrated to have a neoplastic origin, and CSCs have been suggested to transdifferentiate into functional ECs (Rodriguez et al., 2012). It was demonstrated that GBM CSCs contribute to the microvasculature formation by differentiating into ECs *in vitro* and *in vivo,* and that GBM mouse xenografts contain human-derived ECs (Ricci-Vitiani et al., 2010; Wang et al., 2010b; Soda et al., 2011). More recently, vascular pericytes localized near ECs, have been suggested as the actual tumor-derived cells in neovessels, and, by lineage tracing *in vivo,* GBM CSCs have been proposed to be the source of pericytes rather than ECs (Cheng et al., 2013). CXCL12/CXCR4 axis, at least in part, contributes to GBM pericyte formation inducing migration of CXCR4-expressing CSCs toward the perivascular niche, where ECs secrete CXCL12 (Ehtesham et al., 2009; Folkins et al., 2009) and TGF-β drives differentiation into mature pericytes (Cheng et al., 2013). Furthermore, CXCL12 stimulates VEGF secretion in CXCR4-expressing, CD133^+^ CSCs from surgical specimens of human GBM and cell lines, promoting tumor angiogenesis via PI3K/AKT signaling (Ping et al., 2011). Discordant findings have been reported using murine GBM stem-like cells, in which endothelial-like differentiation was associated with CXCL12 expression but CXCL12/CXCR4 blockade did not affect either *in vitro* tube formation or *in vivo* angiogenesis. Thus autocrine/paracrine CXCL12 regulates GBM murine stem cell proliferation but probably not angiogenesis (Uemae et al., 2014).

## TARGETING CXCL12–CXCR4/CXCR7 AXIS IN CANCER: RATIONALE

The notion that CXCR4/R7 expression in cancer is, in most cases, a negative prognostic factor is well supported (Bian et al., 2007; Maderna et al., 2007). CXCL12/CXCR4 axis is involved in tumor development favoring adaptation, survival and proliferation of cancer cells and CSCs in the tumor environment (Scotton et al., 2002; Zhou et al., 2002; Marchesi et al., 2004) and increasing dissemination of CXCR4-expressing tumor cells in response to CXCL12 gradients (Zlotnik et al., 2011) as CXCL12 is markedly expressed in most common sites of metastasis (liver, brain and bone). Moreover, the pro-angiogenic role of CXCR4/R7 and the ability of CXCL12 to up-regulate and synergize with VEGF support the therapeutic relevance of the pharmacological targeting of this pathway. In particular, CXCL12–CXCR4/R7 axis drives hypoxia-dependent angiogenesis and invasiveness of GBM progenitor cells (Ehtesham et al., 2009). CXCR4 also sustains non-pharmacological resistance of tumor cells, through its effects on the stromal microenvironment that supplies growth- and drug-resistance signals to tumor cells (Burger and Kipps, 2006).

Thus specific targeting of CK receptors or CXCL12 itself in cancer management may provide a valuable tool to modulate autocrine/paracrine signaling networks between cancer cells, CSCs and key stromal components (blood vessels, immune cells, fibroblasts), responsible for tumor cell survival, insufficient drug delivery and reduced efficacy of conventional anticancer drugs. CXCR4/R7-based therapeutics might open up the concept of microenvironment targeted therapy as a new pharmacological strategy, to be used in combination with cytotoxic drugs. Indeed, GBM growth and recurrence relies on CSCs that are responsible for tumor vessel formation and bidirectionally interact with tumor ECs *via* secreted factors, including CXCL12, to preserve stemness and promote self-renewal (Stupp et al., 2005; Calabrese et al., 2007; Gatti et al., 2013).

### CXCR4 ANTAGONISTS IN CANCER MANAGEMENT

Most findings on the effects of CXCR4 antagonists in human cancer were obtained in hematological malignancies, disrupting the interactions between CXCR4-expressing leukemia cells and CK secreted by the bone marrow microenvironment. Therefore, results may not be directly translated to solid tumors, but findings could give interesting insights into the potential role of drugs targeting CXCR4, as also shown in tumor animal models.

The discovery of CXCR4 as a co-receptor for T-cell tropic HIV-1 led to the initial development of CXCR4 antagonists such as T140 (Masuda et al., 1992), AMD3100 (De Clercq et al., 1992) and ALX-4C (Doranz et al., 1997). Subsequently, the identification of non-HIV-related functions boosted new applications of CXCR4 inhibitors such as stem cell mobilization, inflammation and cancer treatments.

CXCR4 antagonists can be classified as: (i) modified peptides (T140 and its analogues, BKT140, POL6326, FC131); (ii) small-molecules CXCR4 antagonists (AMD3100, AMD11070, MSX-122, GSK812397); (iii) CXCL12 peptide analogs (CTCE-9908 and CTCE-0214); or (iv) antibodies targeting CXCR4 (MDX-1338/BMS 93656, ALX-0651).

Peptide-based CXCR4 antagonists (TC14012, TZ14001 and TN14003), derived from T140, demonstrated, in preclinical studies, ability to prevent tumor growth and metastasis in animal models of breast, head, and neck carcinoma (Liang et al., 2004; Yoon et al., 2007). In small cell lung cancer cells, TN14003 disrupts CXCR4/CXCL12 interactions and blocks cell adhesion and chemoresistance (Hartmann et al., 2005).

The development of molecules able to inhibit HIV-1/CXCR4 interaction, led to the identification of AMD3100 (De Clercq, 2003), a bicyclam reversible antagonist, the most studied among CXCL12/CXCR4 signaling inhibitors, shown to block CXCL12-mediated calcium mobilization, chemotaxis, and GTP-binding. AMD3100 efficacy in hematopoietic stem cell mobilization was tested in two successful randomized phase III clinical trials on HIV-1 patients (DiPersio et al., 2009) and it was approved by FDA and EMA, in association with G-CSF, for autologous bone marrow transplantation in multiple myeloma (MM) and non-Hodgkin’s lymphoma (NHL)(De Clercq, 2010).

CXCR4 blockade by AMD3100 decreases tumor growth in preclinical GBM models. AMD4365, a novel derivative acting as CXCR4 antagonist inhibits breast tumor formation and reduces lung and liver metastasis (Ling et al., 2013) acting both on tumor and immune cells present in the tumor microenvironment. Association of AMD3100 with bis-chloronitrosourea, showed antitumor efficacy in orthotopic models of GBM demonstrating synergism between CXCR4 inhibition and conventional cytotoxic therapies (Redjal et al., 2006). Currently a combination study with AMD3100 and bevacizumab for patients with recurrent high-grade glioma is ongoing (https://clinicaltrials.gov) with the hypothesis that blockade of CXCR4 will counteract resistance mechanisms to VEGF inhibition. The anti-angiogenic efficacy of AMD3100 was also reported, resulting in a marked reduction of tumor growth and invasiveness in orthotopic GBM-xenotransplanted rats (Ali et al., 2013).

MSX-122, a small molecule identified as a “partial CXCR4 antagonist” (*biased antagonist*) that shows anti-metastatic activity *in vivo* through the unique property of blocking homing and recruitment of cells without mobilizing stem cells (Liang et al., 2012). MSX-122 also inhibits the development of fibrotic process in mice, after radiation-induced lung injury (Shu et al., 2013).

The orally bioavailable derivative AMD11070 powerfully impairs CXCL12/CXCR4-mediated chemotaxis *in vitro* (Mosi et al., 2012; O’Boyle et al., 2013), although phase I/II studies did not warrant further development after safety and pharmacokinetics assessment. BKT140, TG-0054, and POL6326 are currently in clinical evaluation as stem cell mobilizers, for MM, leukemias and lymphomas. The safety and efficacy of BKT140 for mobilization of human CD34^+^ cells in patients with MM has been recently reported (Peled et al., 2014).

CTCE-9908, a CXCL12 modified peptide, beside hematopoietic tumors, is the only CXCR4 antagonist approved analog by FDA for solid tumors, and specifically for the treatment of osteogenic sarcoma. CTCE-9908 inhibits human breast tumor cells growth in mouse xenografts impairing the CXCR4–VEGF loop and lowering tumor VEGF levels (Hassan et al., 2011) and affects breast, prostate and esophageal cancer metastasization in murine models (Wong and Korz, 2008; Richert et al., 2009). Phase I/II clinical trials of this compound, in patients with hepatocellular carcinoma, are currently under evaluation (Wong and Korz, 2008).

The emerging role of CXCR4 in tumor-stroma cross-talk has a great therapeutic potential to deplete minimal residual disease and CSCs: the disruption of CXCR4-mediated tumor cell adhesion to stromal cells might sensitize residual cancer cells and stem cells to standard cytotoxic drugs. In this respect, MDX-1338/BMS 93656, AMD3100 and BKT140 are currently under investigation in phase I/II clinical studies for MM and chronic lymphoid leukemia (CLL).

The development of antibodies against CK receptors has promising therapeutic efficacy, as reported in preclinical and clinical studies. Pharmacological approaches using antibodies exploit a dual mechanism: direct, by selective functional inhibition of the target receptor and, indirect, by potentiation of host immune response through the recruitment of cytotoxic monocytes/macrophages (i.e., antibody-dependent cell-mediated cytotoxicity) or by binding complement factors (i.e., complement-dependent toxicity). Several CK-directed (CCL2, CCL5, and CXCL10) antibodies have been generated and tested in phase I/II clinical trials (Klarenbeek et al., 2012) in both cancer and inflammatory diseases. 30D8, a humanized antibody against mouse/human CXCL12, inhibits tumor growth and/or metastasis and improve arthritis in experimental *in vitro* and *in vivo* models (Zhong et al., 2013). However, the majority of antibodies that successfully entered clinical trials, targets CK receptors rather than ligands. This development was boosted by the identification of the crystal structure of the receptors, particularly concerning the N-terminal extracellular domain, the most accessible region for antibody binding.

A fully-human CXCR4-targeting moAb, MDX-1338/BMS-93656, able to prevent CXCL12 binding, abolished intracellular Ca^2+^ increase and chemotaxis induced by CXCL12 and it is under study to treat relapsed leukemia or, in combination with lenalidomide/dexamethasone or bortezomib/dexamethasone, relapsed/refractory MM (Kuhne et al., 2013).

A new class of antibody-derived therapeutics, based on single-domain heavy-chain (VHH) antibody fragment, named nanobodies, displays high stability, low toxicity and antigen-binding capacity. The first CK receptor targeted nanobody against CXCR4 (Jahnichen et al., 2010), showed 100-fold higher affinity than AMD3100. CXCR4 nanobodies completely inhibit entry of CXCR4-tropic HIV-1 strains *in vitro* and intravenous injection mobilizes stem cell in animal models, acting similarly to AMD3100 (Jahnichen et al., 2010). Currently, a CXCR4-inhibiting nanobody, ALX-0651, is in phase I trial (https://clinicaltrials.gov:NCT01374503).

Recent insights in structural features of both CXCR4 and CXCL12 and structure-activity relationships, improved chemical modeling and structure-based development of candidate molecules to be screened as CXCR4-antagonists (Wu et al., 2010). A virtual screening of the National Cancer Institute’s Open Chemical Repository Collection, using a homology model of CXCR4, led to the identification of a lead structure (Kim et al., 2012). Furthermore, a new family of CXCR4 modulators, as phianidine A, identified by screening a library of marine compounds using a simple pharmacophoric model identified after CXCR4 crystal structure, was recently reported (Vitale et al., 2013).

### CXCR7 ANTAGONISTS

In addition to CXCR4, CXCR7 represents a viable target for anticancer and antimetastatic drugs. Inhibition of CXCR7 with selective antagonists in mice engrafted with breast and lung cancer cell lines and experiments testing overexpression or silencing of CXCR7 in tumor cells, collectively support the idea that CXCR7 promotes tumor growth (Miao et al., 2007).

CXCR7 antagonists are expected to act mainly by reducing tumor cell extravasation and thus metastasis, and blocking tumor angiogenesis, as demonstrated by CCX771, a synthetic CXCR7 ligand markedly more potent at inhibiting transendothelial migration than AMD3100. It also stimulates β-arrestin recruitment to CXCR7 in a lymphoblastic leukemia model (Zabel et al., 2009). Moreover, exposure of CXCR4^+^CXCR7^+^ cancer cells to CXCL12 greatly enhances migration of human Burkitt’s lymphoma cells through a human HUVEC endothelial cell monolayer as *in vitro* model of transendothelial migration (Zabel et al., 2011) suggesting the potential efficacy of CXCR7 antagonists in blocking CXCL12-mediated metastatic spread of CXCR4^+^CXCR7^+^ tumor cells, *in vivo*.

CXCR7 is also an attractive therapeutic target for hematopoietic stem cell (HSC) mobilization-inducing agents since its expression might be necessary to direct HSCs to the niches sustaining their capacity of migration. Although CXCR7 regulation of BMSC niche has not been completely defined, it should represent a relevant goal for research since the possible inclusion of CXCR7 antagonists in the current formulation of HSC mobilizers (granulocyte-colony-stimulating factor, G-CSF, plus AMD3100) might reduce the percentage of patients in which that mobilization protocol fails (To et al., 2011).

In order to fully elucidate the complex pharmacology and potential therapeutic utility of CXCR7 receptor antagonists, CXCR7 structural models would be highly useful. However, these detailed structures are currently not available. At present, a limited number of CXCR7 ligands have been reported (Kalatskaya et al., 2009; Gravel et al., 2010; Wijtmans et al., 2012), therefore the application of GPCR homology modeling and virtual screening, previously used in CXCR4 studies, for novel CXCR7 ligand identification represents a promising tool (Yoshikawa et al., 2013). AMD3100 and the peptidomimetic CXCR4 antagonist TC14012 have also been reported to act as partial CXCR7 agonists (Kalatskaya et al., 2009; Gravel et al., 2010). Several pharmacological studies with small-molecule CXCR7 antagonists endowed with reasonable affinities have been reported, but none disclosed a structure for the antagonists (Burns et al., 2006; Zabel et al., 2009; Hattermann et al., 2010; Rajagopal et al., 2010; Cruz-Orengo et al., 2011). A recent paper describes the first reported combined synthetic, modeling and pharmacological effort on small molecules targeting CXCR7 (Wijtmans et al., 2012).

### DUAL TARGETING OF CXCR4-R7 OR CXCL12 BLOCKADE

Since CXCR4 and CXCR7 are both involved in cancer malignancy, and in particular in GBM angiogenesis, molecules able to interact and block either CXCL12 itself or both receptors simultaneously could represent an improved pharmacological approach (Duda et al., 2011; Singh et al., 2013).

However, as far as dual receptor binding the current available data are rather complex. Some CXCR4 or CXCR7 antagonists were reported to bind also the other receptor although not always acting as antagonist, but partial agonist activity was reported. This is also the case for the prototype CXCR4 antagonist AMD3100 that may act as CXCR7 partial agonist (Kalatskaya et al., 2009). Moreover, CXCR7 agonists selectively activating β-arestin were shown to down-regulate CXCR4 (Uto-Konomi et al., 2013). This differential modulatory effect on the receptors might induce complex biological responses according to the cell analyzed. As far as GBM it was reported that CSCs mainly express CXCR4 while CXCR7 is mainly located in differentiated cells and endothelia (Hattermann et al., 2010; Gatti et al., 2013). In other models the receptors are co-expressed, acting also as heterodimers. Thus, the potential synergism induced by ligands with dual specificity has to be evaluated in the specific cell context and in relation to the agonist/antagonist properties of the molecule.

A more defined picture is obtainable blocking the activity of both CXCR4 and CXCR7 interfering with their ligand. Indeed, synthetic compounds from the family of chalcones, able to bind to CXCL12 with high affinity to prevent its binding to the receptors, have been reported to inhibit inflammatory responses in eosinophils (Hachet-Haas et al., 2008).

Moreover, NOX-A12, an RNA oligonucleotide that binds and neutralizes CXCL12 with high affinity (Liang et al., 2007), is currently in clinical trial for leukemia and MM, displaying antineoplastic activity and stem cell-mobilization from bone marrow. NOX–A12 interferes with CLL cell motility and BMSC-mediated drug resistance, sensitizing CLL cells towards bendamustine and fludarabine, in BMSC co-cultures. Noteworthy, NOX-A12 has been recently reported to be effective in inhibiting or delaying recurrences following irradiation in an *in vivo* GBM model (Liu et al., 2014).

## CONCLUSION

In recent years, molecularly targeted drugs have joined conventional chemo- and radio-therapies for the management of several cancers, and have become the first-line treatments for tumors lacking efficacious therapeutic options, such as the approval of bevacizumab for recurrent GBM. Benefits of targeted therapy in terms of overall survival are modest, however in GBM, whose median survival is approximately 15 months, even an improvement of progression-free survival could be encouraging. In this context, the blockade of CXCR4/R7 signaling represents an alternative or additional target for neo-adjuvant treatments. However, a better understanding of the biology of the CK receptors and ligands in CSCs, GBM tissue and stroma, is needed to clarify their role in tumorigenesis and define the actual best therapeutic target among stromal cells, CSC and differentiated cancer cells or their whole cross-talk. In addition, since CXCR4 and CXCR7 are involved in angiogenesis, targeting this chemokinergic system could improve the poor efficacy of inhibitors of angiogenesis in several cancers including GBM.

Conceivably, the combination of CXCR4 and CXCR7 antagonists could represent powerful tool to reduce tumor cell invasion and metastasis. Moreover, the role of CXCL12 pathway in tumor resistance, acting both directly, to promote cancer cell and CSC survival and angiogenesis, and indirectly, to recruit stromal cells that through paracrine activity induce recurrence and metastasis, is crucial for cancer therapy.

However, development of preclinical and translational research targeting microenvironment in hematopoietic and solid malignancies should be paralleled by solution of its limitations as the actual benefit of combination with cytotoxic agents, duration (length) of responses and potential development of mechanisms of resistance. Moreover, each cancer type might require a different CXCR4 antagonist, exploiting pharmacological features such as oral availability and pharmacokinetics, and the prevalent ability to mobilize hematopoietic cells or to inhibit metastasis or invasion of cancer cells.

## Conflict of Interest Statement

The authors declare that the research was conducted in the absence of any commercial or financial relationships that could be construed as a potential conflict of interest.
